# Supplementary material for: Protein SUMOylation promotes cAMP-independent EPAC1 activation
Source: Cell Mol Life Sci. 2024 Jul 4;81(1):283. doi: 10.1007/s00018-024-05315-y (PMC11335207; doi:10.1007/s00018-024-05315-y)
Supplement: Supplementary file 1 — Supplementary file1 (DOCX 10856 KB) [file 18_2024_5315_MOESM1_ESM.docx]

**Protein SUMOylation promotes cAMP-independent EPAC1 activation.**

Wenli Yang^a,b,c, #^, Fang C. Mei^a, b, c, #^, Wei Lin ^a, b, c^, Mark A. White^d^, Li Li^c^, Yue Li^a,§^, Sheng Pan^a, c^, and Xiaodong Cheng^a, b, c, *^

^a^Department of Integrative Biology and Pharmacology, ^b^Texas Therapeutics Institute, ^c^Brown Foundation Institute of Molecular Medicine, The University of Texas Health Science Center, Houston, Texas, USA. ^d^Department of Biochemistry and Molecular Biology, Sealy Center for Structural Biology and Molecular Biophysics, The University of Texas Medical Branch at Galveston, Galveston, Texas, USA.

^#^These authors contributed equally.

^§^Current Address: Cell Therapy Manufacturing Center. 2130 W Holcombe Blvd, Houston TX, 77030.

*Corresponding author: Xiaodong Cheng.

**Email:**  xiaodong.cheng@uth.tmc.edu

**Keywords:** SUMO, SUMO-interacting motif, Biomolecular condensate**,** Heat shock Molecular dynamics

**Supplementary Data**

**Table S1. Predicted SUMOylation sites and SUMO-interacting motifs.**

| **Putative SUMOylation site** | | |
| --- | --- | --- |
| **Position** | **Sequence** | **Database Hit** |
| K212 | KAVAHLSNSVKRELAAVLLFE | 13 |
| K310 | FNRIIKDVEAKTMRLEEHGKV | 1 |
| K319 | AKTMRLEEHGKVVLVLERASQ | 1 |
| K517 | GSSCAIQVGDKVPYDICRPDH | 3 |
| K561 | DGWTKGQVLVKVNSAGDAIGL | 1 |
| K698 | PRAQLLRKFIKLAAHLKEQKN | 2 |
| K764 | NHRVYRLALAKLSPPVIPFMP | 1 |
| K778 | PVIPFMPLLLKDMTFIHEGNH | 1 |
| K864 | ASTWAYVQQLKVIDNQRELSR | 1 |
| **Putative SUMO-interacting motif** | | |
| **Position** | **Sequence** | **Score/Database Hit** |
| AA 320-323 | MRLEEHGKVVLVLERASQGA | 2.020/2 |
| AA 321-324 | RLEEHGKVVLVLERASQGAG | 3.166/0 |


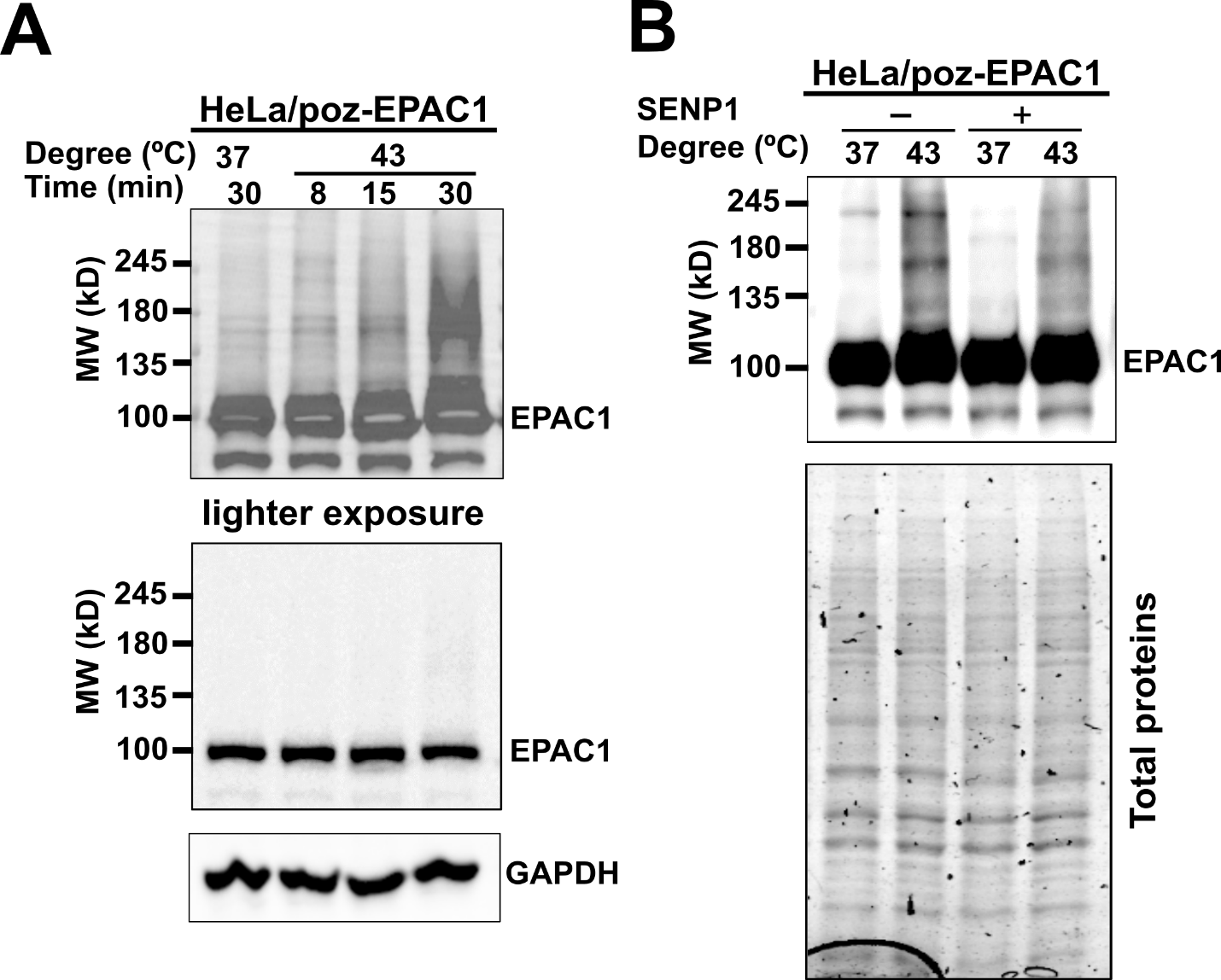


**Figure S1. Heat shock promotes EPAC1 post-translational modifications (PTM).** (A) Levels of cellular EPAC1 PTM, probed by immunoblotting using anti-EPAC1 antibody, in HeLa/poz-EPAC1 cells in response to heat shock as a function of time. (B) Levels of cellular EPAC1 PTM in HeLa/poz-EPAC1 cells probed by immunoblotting using anti-EPAC1 antibody, with and without heat shock (30 min) and with or without SENP1 (220 nM) treatment at 37 °C for 20 min. Similar results were obtained from at least three independent experiments.


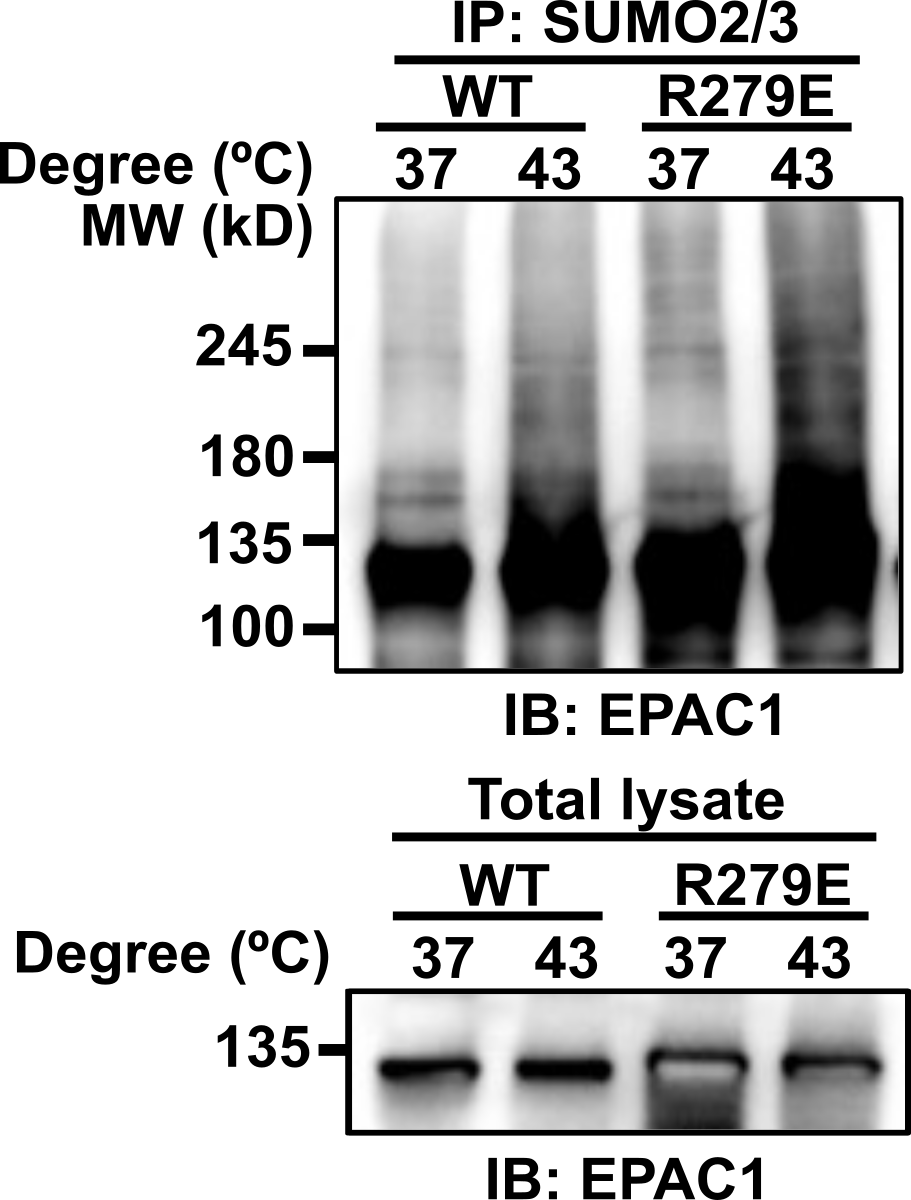


**Figure S2. Heat-shock induces SUMOylation of EPAC1.** Anti-EPAC1 immunoblotting of SUMO2/3 affinity pull-down of cell lysates derived from HEK293/EPAC1-APEX2 and HEK293/EPAC1(R279E)-APEX2 cells after incubation at 37 or 43 °C for 30 min.

.


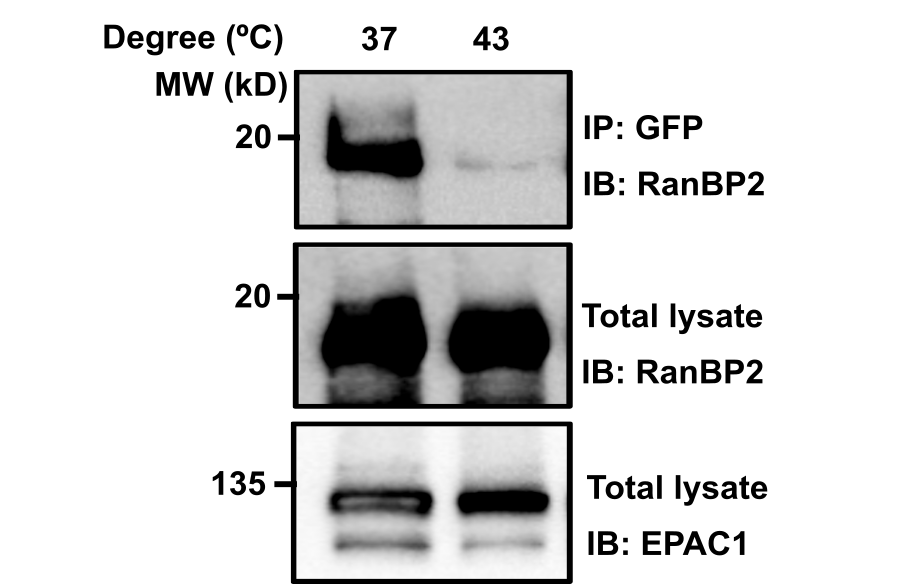


**Figure S3. Heat shock reduces EPAC1 RanBP2 interaction.** Interaction between ectopically expressed EPAC1-EYFP and endogenous RanBP2 in response to heat shock probed by affinity purification using anti-GFP antibodies.

**
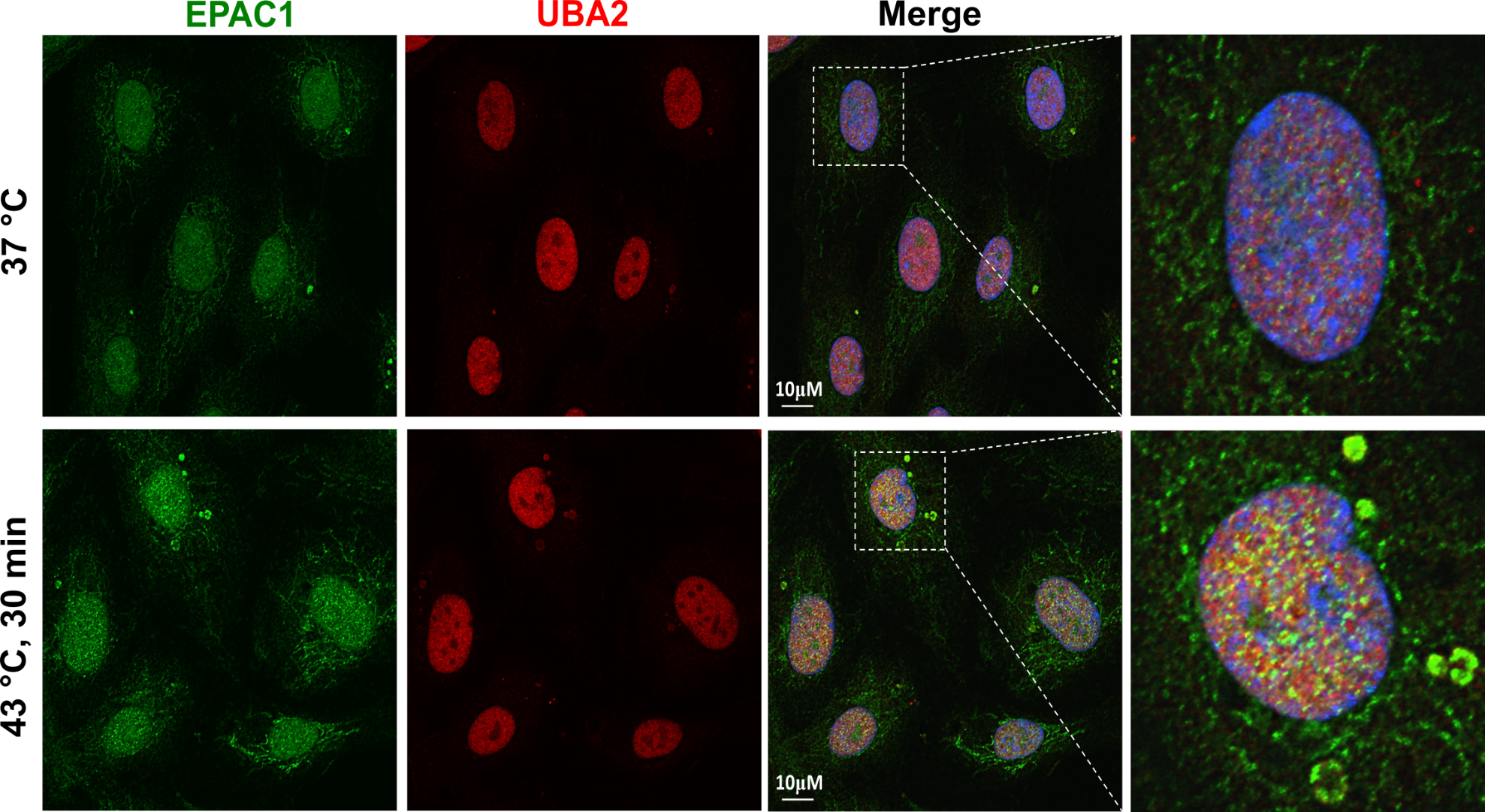
**

**Figure S4. Heat shock-induced formation of endogenous nuclear EPAC1 and UBA2 condensates.** Confocal images of HUVECs stained for endogenous EPAC1 (green) and UBA2 (red) in response to heat shock treatment at 43 °C for 30 min. Bar = 10 μm.

**
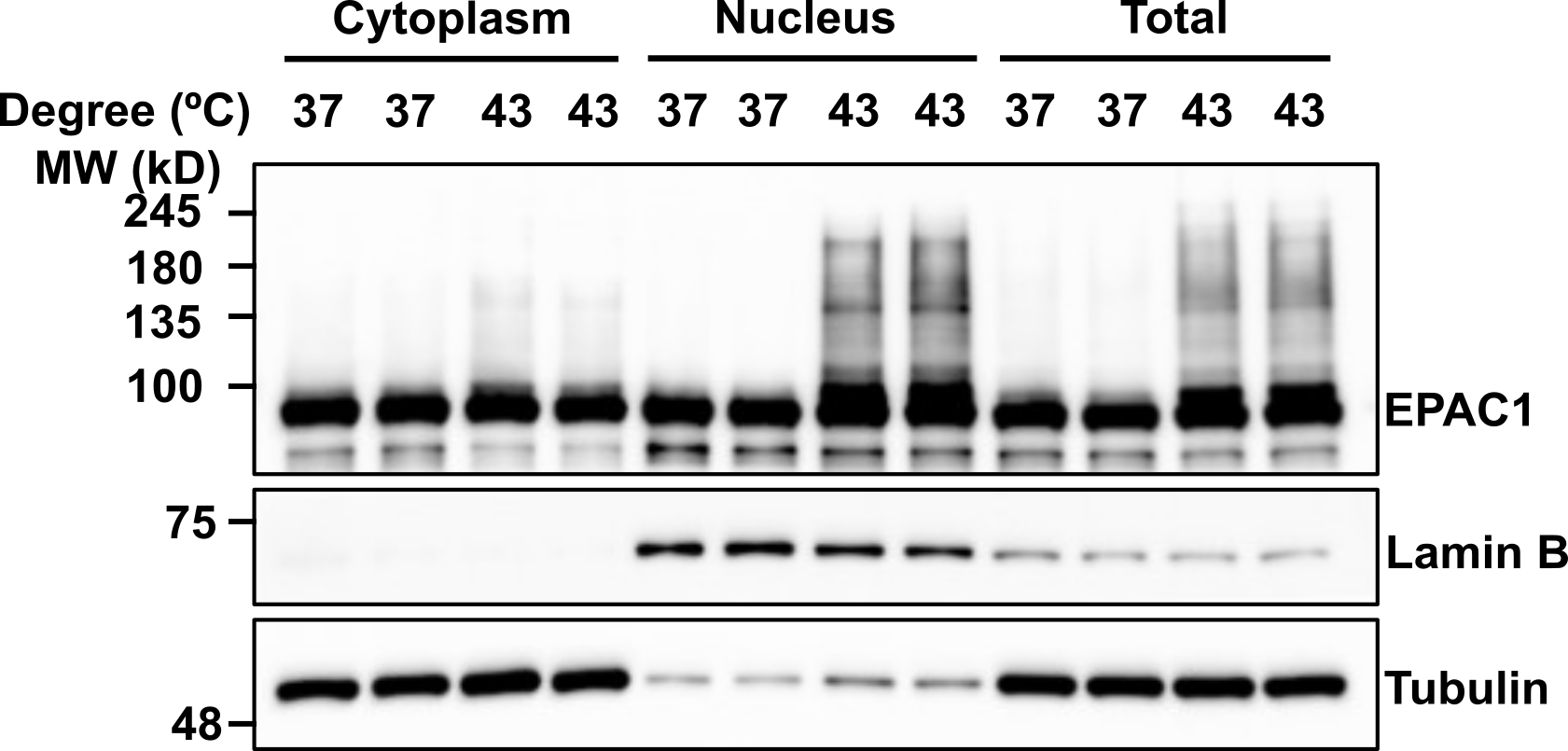
**

**Figure S5. Cytosolic and nuclear EPAC1 PTM in response to heat shock.** Levels of total, cytosolic, and nuclear EPAC1 PTM in HEK293/EPAC1-Flag cells probed by immune-blotting using anti-EPAC1 antibody in response to heat shock treatment at 43 °C for 30 min.

**
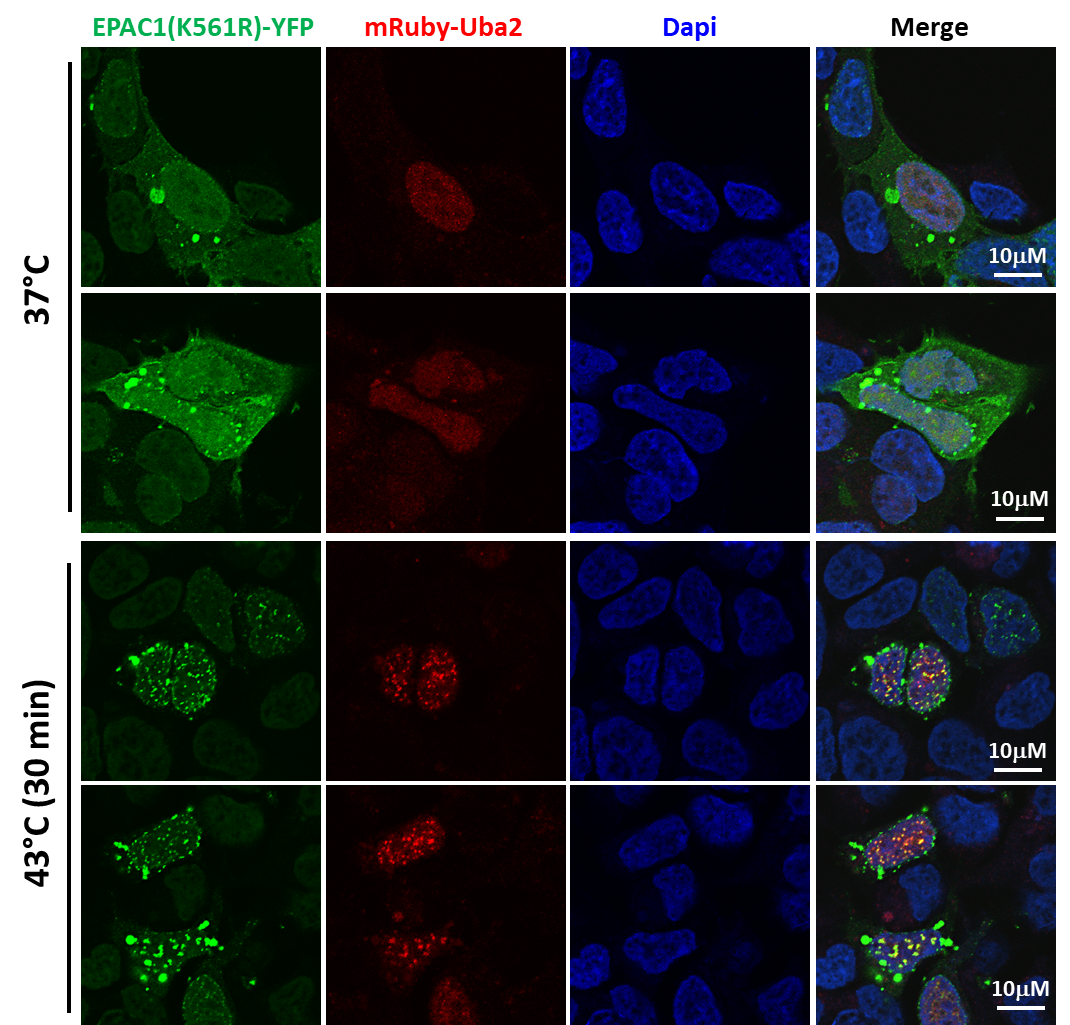
**

**Figure S6. Heat shock-induced formation of nuclear EPAC1(K561R)-EYFP/mRuby-UBA2 condensates.** Confocal images of HEK293 cells ectopically expressing EPAC1(K561R)-EYFP and mBuby-UBA2 in response to heat shock treatment at 43 °C for 30 min. Bar = 10 μm.

**
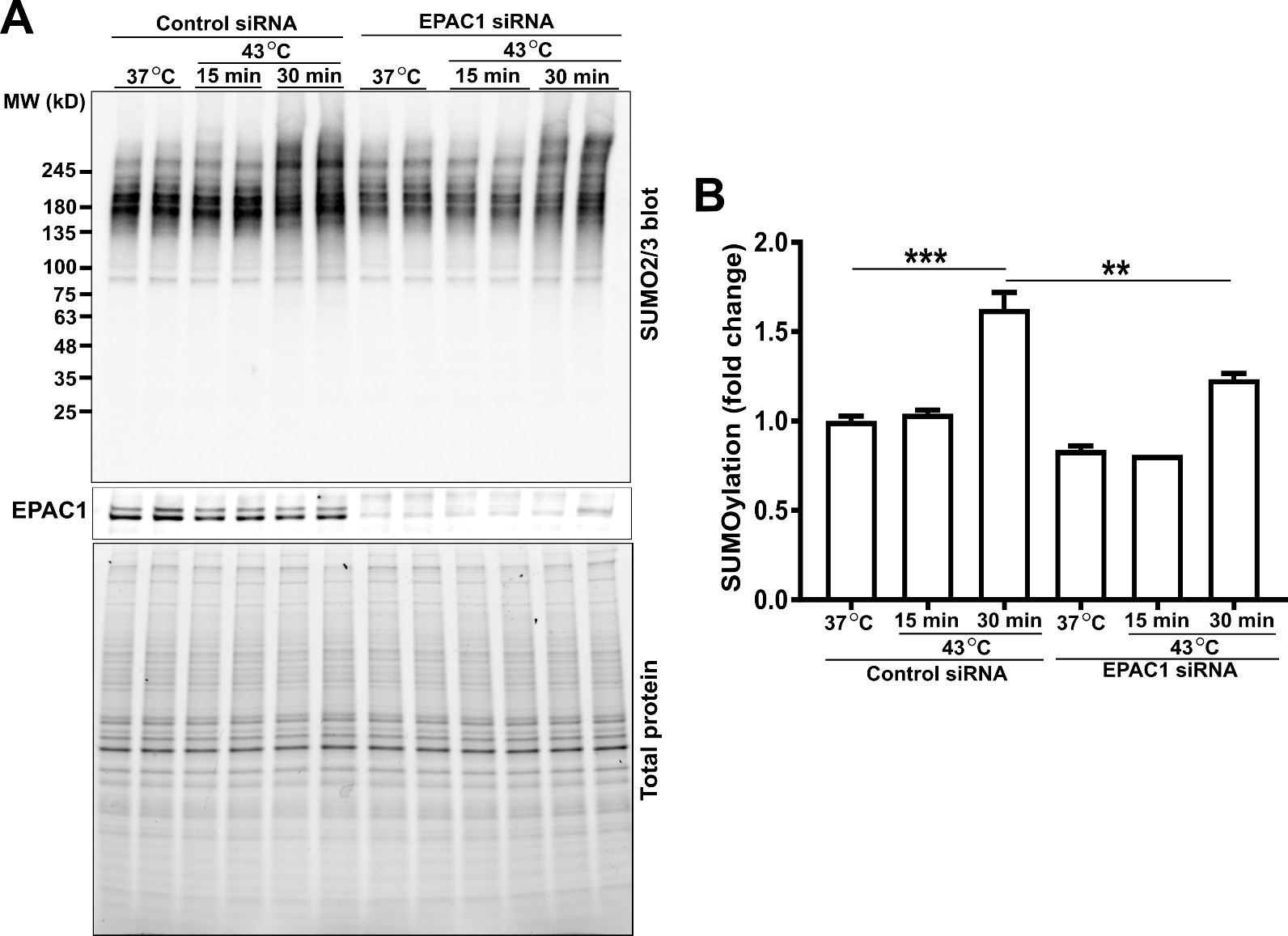
**

**Figure S7. Role of EPAC1 in heat shock-induced cellular SUMOylation.** (A), Levels of cellular SUMOylation in HUVECs transfected with control or EPAC1-specific siRNA in response to heat shock treatment (43 °C, 15 or 30 min). (B), Quantification of heat shock-induced cellular SUMOylation in HUVECs (N = 4).


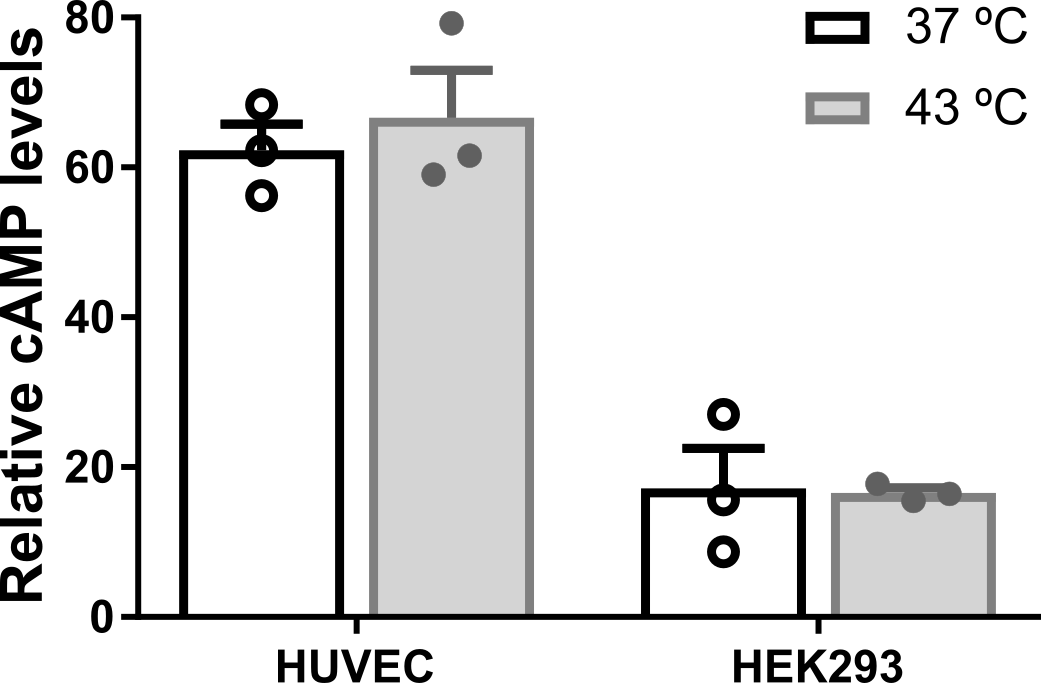


**Figure S8. Effects of heat shock on levels of intracellular cAMP.** Levels of cellular cAMP in HUVEC and HEK293 cells at 37 °C or after heat shock at 43 °C for 30 min.

**
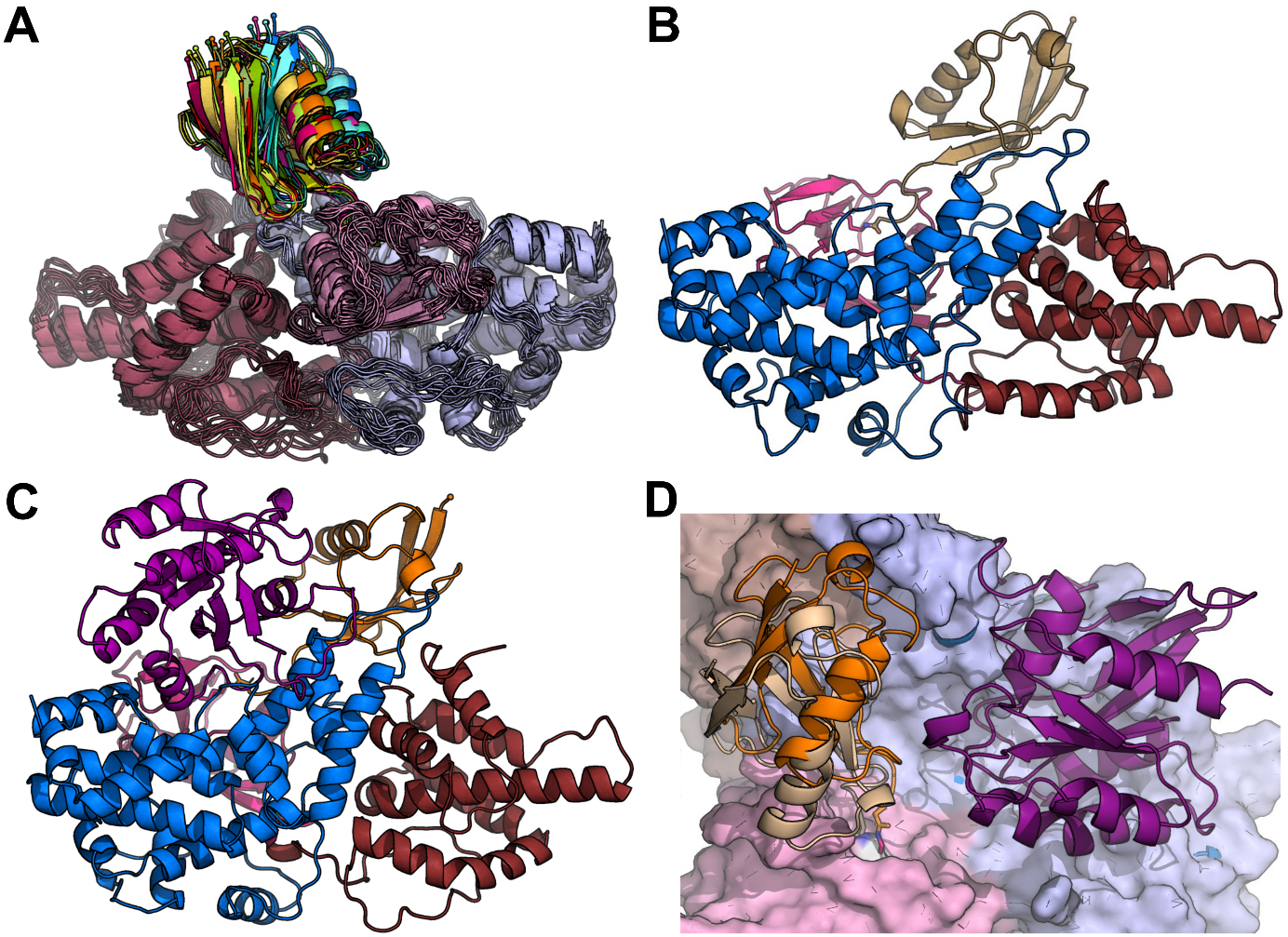
**

**Figure S9. The EPAC1^CT^-SUMO:Rap1 and EPAC1^CT^-SUMO models after 30 ns of Molecular Dynamics.** (A) SUMO orientations EPAC1^CT^-SUMO during the 30 ns MD. The EPAC1^CT^-SUMO MD model is colored by domain: REM: brown, RAP: pink, and GEF: blue. The SUMO is shown as a heat map colored by MD time from blue (0) to red (30 ns). (B) The final EPAC1^CT^-SUMO MD model, with the SUMO colored in beige. (C) The final EPAC1^CT^-SUMO:Rap1 MD model, with the SUMO colored in orange and Rap1 colored in purple. (D) SUMO orientation shifts slightly in the absence of the Rap1, but it remains in roughly the same position. The EPAC1^CT^-SUMO MD model is shown in light tones with the SUMO colored in beige, and EPAC1 surfaced, while the EPAC1^CT^-SUMO:Rap1 MD model is in bold tones with the SUMO colored in orange and Rap1 colored in purple.

**
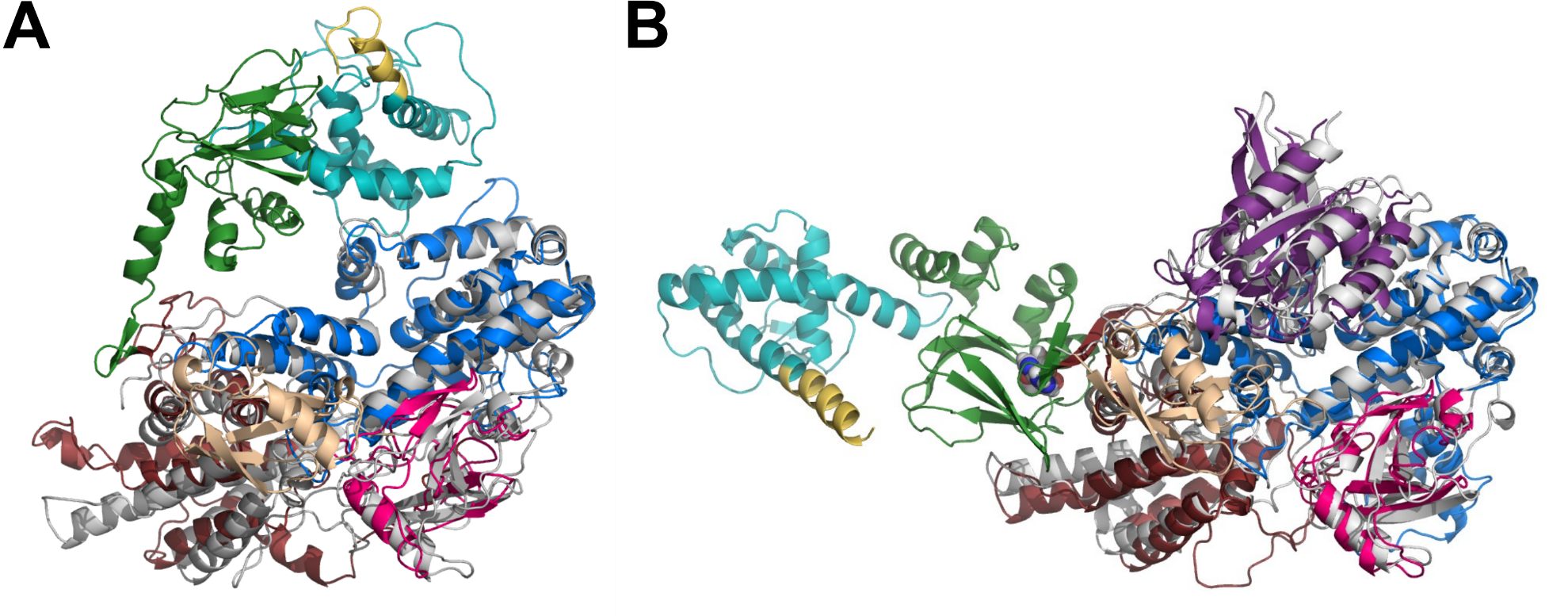
Figure S10. The MD models of EPAC1^CT^-SUMO and EPAC1^CT^-SUMO:Rap1.** (A) The EPAC1^CT^-SUMO model with EPAC^CT^ colored in grey and SUMO colored in beige superimposed with the apo-EPAC1 model (DEP: cyan; CNBD: green; REM: brown; RA: pink; CDC25HD: blue). (B) The EPAC1^CT^-SUMO:Rap1 model with EPAC^CT^ colored in grey, SUMO colored in beige, and Rap1 colored in purple, superimposed with the ternary EPAC1:cAMP:Rap1 model.
